# Collection of a Continuous Long-Term Dataset for the Evaluation of Wi-Fi-Fingerprinting-Based Indoor Positioning Systems

**DOI:** 10.3390/s22228585

**Published:** 2022-11-08

**Authors:** Ivo Silva, Cristiano Pendão, Adriano Moreira

**Affiliations:** Algoritmi Research Centre/LASI, University of Minho, 4800-058 Guimarães, Portugal

**Keywords:** Wi-Fi dataset, Wireless Sensor Network, indoor positioning, Wi-Fi fingerprinting, radio maps, long-term dataset, dataset, validation

## Abstract

Indoor positioning and navigation have been attracting interest from the research community for quite some time. Nowadays, new fields, such as the Internet of Things, Industry 4.0, and augmented reality, are increasing the demand for indoor positioning solutions capable of delivering specific positioning performances not only in simulation but also in the real world; hence, validation in real-world environments is essential. However, collecting real-world data is a time-consuming and costly endeavor, and many research teams lack the resources to perform experiments across different environments, which are required for high-quality validation. Publicly available datasets are a solution that provides the necessary resources to perform this type of validation and to promote research work reproducibility. Unfortunately, for different reasons, and despite some initiatives promoting data sharing, the number and diversity of datasets available are still very limited. In this paper, we introduce and describe a new public dataset which has the unique characteristic of being collected over a long period (2+ years), and it can be used for different Wi-Fi-based positioning studies. In addition, we also describe the solution (Wireless Sensor Network (WSN) + mobile unit) developed to collect this dataset, allowing researchers to replicate the method and collect similar datasets in other spaces.

## 1. Introduction

The publication of results obtained through simulation or in small-scale experiments, usually in small office spaces or laboratories, has been the trend in indoor positioning publications. The process of conducting large-scale validation in the real world is costly and time-consuming; therefore, many research teams lack the necessary resources to prepare the setup and collect all the necessary data, including ground truth information.

Simulation and synthetic data are valuable assets in the initial stages of research and development. This type of data can be used to identify and mitigate potential events that can degrade the positioning performance. However, real-world data are essential to measure the actual performance of the system in a complex scenario, since simulation cannot fully mimic all the characteristics and effects found in a real environment.

The need for comprehensive validation is increasing with emerging technologies and fields of application, which require solutions that can deliver good position performance not only on paper but also in the real world. Industry 4.0 is one of the areas where indoor positioning systems will play an important role and where performance requirements (e.g., accuracy, precision, scalability and reliability) must be ensured before deployment.

Public available datasets are a key component in the development and validation processes allowing one to: promote reproducibility and transparency; promote a direct comparison between competing solutions; evaluate a solution in multiple environments; mitigate the performance degradation in specific cases or events, which is difficult to simulate.

In areas such as machine learning, sharing datasets is a well-established process for validation and comparison of results. In the indoor positioning field, some initiatives, such as the International Conference on Indoor Positioning and Indoor Navigation (IPIN) Competition (https://evaal.aaloa.org/2022/call-for-competitions (accessed on 6 June 2022)), are actively promoting dataset sharing. The IPIN competition is based on the EvAAL framework, which was conceived with the purpose of evaluating active and assisted living positioning systems through competitive benchmarking [1]. Unfortunately, most authors still do not share their datasets for different reasons, for instance: collecting a dataset is time-consuming, as reported in many works [2,3,4], since it requires the mapping of reference points, data collection, and post-processing; the main focus of research teams is to develop novel positioning and localization methods, so they use their own datasets for developing and testing their systems, without the objective of sharing the datasets with the community; due to privacy reasons [5,6], since these datasets comprise Wi-Fi information, including Service Set Identifier (SSID) and Media Access Control (MAC) addresses of Access Points (APs).

Most Wi-Fi-based datasets, e.g., [7,8,9,10,11], are collected for Wi-Fi fingerprinting, which include training and test data. Both sets of data are usually composed of Wi-Fi samples that were collected at a set of known locations (Reference Points (RPs)), within a very limited time span. It is well known that Wi-Fi signals suffer significant variation in indoor environments [12,13], even during short periods of time, so most Wi-Fi datasets do not accurately represent the radio environment shortly after they are collected. This is why the radio map should be kept up to date to provide the best possible results with Wi-Fi fingerprinting.

The main benefit of continuously monitoring the radio environment is that it allows a continuous perception of the Wi-Fi signals over time. This can be explored to keep the radio map updated, or to detect anomalous events that cause interference or other problems in the radio environment. In order to continuously monitor the radio signals of an indoor environment over time, it is necessary to deploy a network of devices in the building to collect these signals over time, which can be implemented with a Wireless Sensor Network (WSN).

This paper introduces a new publicly available dataset that can be used for research and development of Wi-Fi-based indoor positioning solutions. Some of the key characteristics of this dataset are: data span a long period (over 2 years); the data were obtained automatically from a WSN of several Monitoring Devices (MDs) deployed in the building, which continuously collected Wi-Fi samples; to complement the automatic and continuous long-term dataset, multiple site surveys were conducted by manually collecting Wi-Fi samples in many RPs, spanning over 2 years. As far as we know from our research on this topic (in Section 2), no other available dataset combines these characteristics. Moreover, to avoid privacy issues, measures were taken for data anonymization, such as the replacement of MAC addresses of the APs (also known as Basic Service Set Identifier (BSSID)). The main motivation for collecting this dataset was to conduct a study to understand how and why the radio maps degrade over time, considering a long period of time to have a perception of the radio environment during the collection period [14].

The main contributions in this work are summarized as follows. The first is the dataset itself, which has been provided to the research community. The second is the documentation of the dataset, including the full description of the data, and the scripts to parse and process the data and create plots to analyze data, which can be adapted for other use cases and to analyze other aspects of the data. The third contribution is the full description of the experimental setup and method used to automatically collect the dataset over a long time, allowing other researchers to replicate the setup and collect data in other spaces. We also provide some statistical analysis of the data.

This dataset is useful for further research performing different types of analyses and studies. For example:

1. Analysis of long-term variations and assessment of long-term radio map degradation. This dataset was used for this purpose in [14].
2. Devising new solutions to overcome radio map degradation. Significant efforts have been made in the past to develop methods to keep the radio maps up to date using interpolation, e.g., considering techniques such as Log-distance Path Loss (LDPL) [15,16,17], Radial Basis Functions (RBF) [18], Inverse Distance Weighted (IDW) [19,20], Voronoi tesselation [21], and Kriging [22,23].
3. Monitoring the radio environment for anomaly detection [24,25,26] and to detect trends in data, similarly to what has been done in other research [27].
4. Testing and evaluation of an Indoor Positioning System (IPS) or for benchmarking and performance comparison between different IPSs.

This paper is organized as follows: the related work is introduced in Section 2; in Section 4 a description of the experimental setup is given, including the building where the data collection was performed and the software and hardware used to collect the dataset; Section 5 presents the description of the database, including the file and folder structure, and the data formats; an overview of the long-term dataset is given in Section 6, along with a data analysis; example applications where this dataset was used are described in Section 7; finally, the conclusions are discussed in Section 8.

## 2. Related Work

In indoor environments, several technologies may be used for enabling indoor localization, e.g., Wi-Fi [15], Ultra Wide-band (UWB) [28], and Bluetooth Low Energy (BLE) [29]. These technologies are used as alternatives to Global Navigation Satellite System (GNSS), whose performance is quite limited in indoor environments, due to the lack of direct line-of-sight, since the buildings block the satellite signals.

Wi-Fi has been explored for localization of pedestrians [30], mobile robots [31], industrial vehicles [32], etc. It is one of the most used technologies for indoor localization due to several reasons: (1) it is widespread and available in most buildings; (2) it is low-cost, since it takes advantage of the already available infrastructure; (3) most devices (smartphones, laptops, smartwatches, printers, etc.) have a Wi-Fi interface—thus, they can be localized using Wi-Fi as the supporting technology; (4) it is a versatile technology that supports many localization techniques, e.g., fingerprinting (scene analysis) [15,33], deep learning [34], angle of arrival [35], time of flight [36] and time difference of arrival [37].

Many Wi-Fi-based IPSs explore Wi-Fi fingerprinting [15,33,38], a technique that relies on a radio map used to estimate a position. Due to the characteristics of radio signals indoors being affected by propagation effects (reflection, refraction, absorption, etc.) and interference, among others, radio maps tend to degrade [14] over time. However, this degradation is not gradual; it is affected by significant changes in the Wi-Fi infrastructure, so the right time to update the radio map can be difficult to identify. This is one of the main reasons why we performed this long-term data collection and now share this dataset with the research community, so that it may be used for other research, e.g., anomaly detection.

To test and validate their IPS, researchers usually collect their datasets in the scenario where they will be conducting experiments. The reported performance of the system is typically obtained from those datasets. Although this enriches the research and helps with validating the proposed IPS, researchers normally do not share these datasets; therefore, it is not possible to directly compare the performances of these systems using the same data or replicate their results. The IPIN Competition (Track 3) [39,40] has been contributing to overcoming this problem. Track 3 is an off-site competition, where datasets with smartphone sensor data are shared with competitors, and the competing IPSs are directly compared using the same test dataset. Since 2015, the datasets from IPIN’s Track 3 Competition have been available at (http://ipin-conference.org/resources.html (accessed on 6 June 2022)).

Several Wi-Fi datasets [7,8] have been published, aiming to increase the publicly available datasets that can be used by researchers for testing their IPSs based on Wi-Fi fingerprinting. More recently, a long-term dataset [9,10,11] was published, including data from several manual site surveys that were performed over a period of 25 months. The dataset introduced in this paper has distinct characteristics in comparison to this one. First, data were collected continuously, including data from several MDs in fixed locations. Second, it also includes data from several manual site surveys which complement the continuous long-term data. Third, it contains data collected for a period of over 2 years.

We searched Zenodo (https://zenodo.org/ (accessed on 12 July 2022)) with the "indoor AND positioning AND Wi-Fi" terms and found seven datasets that matched the search terms. Upon a more specific search for "indoor AND positioning AND Wi-Fi AND long-term", only one match was found—namely, the already mentioned long-term dataset [11]. Therefore, with the publication of this dataset, we intend to contribute to increasing the number and diversity of datasets publicly available for the R&D of Wi-Fi-based solutions. We hope that this contribution may encourage others to also share their datasets.

## 3. Approach for Dataset Collection

The purpose of collecting this long-term dataset was to study the radio environment over time to understand how it varies affects the positioning performance of a Wi-Fi-based IPS. When considering the collection of a long-term dataset for Wi-Fi-based positioning systems, we considered several requirements:

- Sample rate: Time between consecutive Wi-Fi samples. It should be sufficient to measure the short-term and long-term variations in the radio environment. Signal strength values may register significant variation between consecutive scans, which can hinder the performance of an IPS [13,41].
- Survey points: Ideally, the collection should be done in as many points as possible to have a higher spatial resolution in the collected data. However, that is not possible, so the distribution of MDs should maximize the coverage area of the building.
- Time span: The data collection should be long enough to observe infrastructure changes (addition or removal of APs that compose the building’s Wi-Fi infrastructure) that may occur after months or even years [14]. Having a long-term dataset allows one to analyze trends in the short term and long term, e.g., whether there are patterns that occur weekly or monthly, or whether there is a month when radio signals change a lot.
- Wi-Fi scans: Nowadays, the Wi-Fi APs deployed in indoor environments support IEEE 802.11 b/g/n (2.4 GHz frequency band) and IEEE 802.11 a/ac/ax (5 GHz frequency band); hence, the Wi-Fi scans should include APs emitting in the 2.4 and 5 GHz frequency bands. Consequently, this dataset is of high quality, as it better represents the radio environment.
- Indoor Positioning: The collected data should include calibration and testing datasets with ground truth, which can be used to validate and test IPSs.
- Data collection: A continuous long-term dataset should be collected autonomously by deploying MDs in known locations, which allows data to be collected without human intervention. Manual site surveys should be performed to collect data in known locations, to be used in positioning applications considering a realistic scenario, e.g., a pedestrian holding a smartphone or a Wi-Fi-enabled indoor vehicle.
  In the following sections, we describe how we addressed these challenges in the system that was developed to collect the dataset.

## 4. Experimental Setup

**Figure 1:**
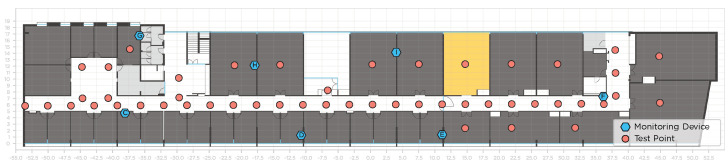
Floor plan of the building, including the positions of the Monitoring Devices and the site survey Reference Points.

The data collection was performed in the Department of Information Systems at the Azurém Campus of the University of Minho (Figure 1). The building comprises several offices, laboratories, and classrooms; hence, it is frequented daily by students, professors, and researchers. Wi-Fi access to students and faculty staff is provided by the University’s Wireless Local Area Network (WLAN), composed of several APs distributed through the building, all emitting the same network SSID. Despite that, many other APs from other networks are detected in the building.

Seven fixed devices, which we refer as MDs, were installed in the building in known locations (blue hexagons in Figure 1), forming a WSN, continuously collecting Wi-Fi samples. The RPs selected for the site surveys (salmon circles in Figure 1) were initially mapped on the building’s floor plan (in OSM format). Then, these points were physically marked on the floor of the building. We used a measuring laser to measure the distances to reference walls and building beams, which allowed an accurate mapping of these points in the real world.

**Figure 2:**
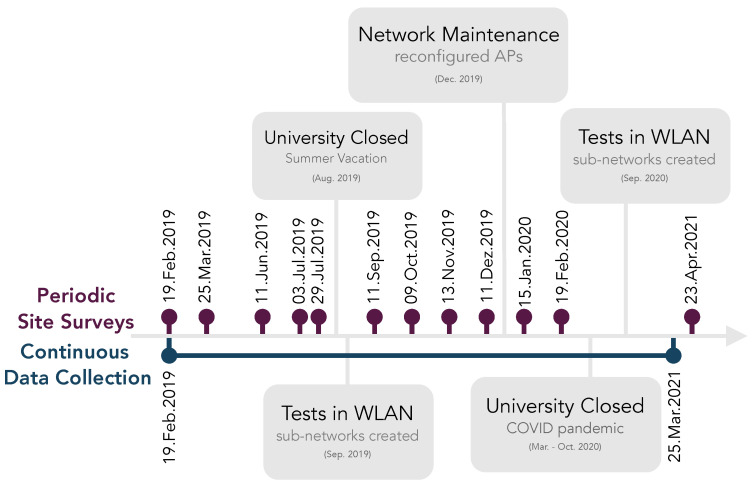
Timeline of long-term data collection and periodic manual site surveys.

Figure 2 depicts the timeline of the data collection, considering that the data were collected in two distinct ways. The first was using a set of MDs that compose a WSN to automatically collect data in the long-term, as represented by the blue line in Figure 2. The second was by periodically conducting manual site surveys using a mobile unit moved across many RPs, whose dates are represented by the purple markers in Figure 2. The remainder of this section describes the solution for long-term data collection, including the hardware and software.

### 4.1. Hardware

The hardware part of the experimental scenario is described in this section, including the Raspberry Pi device that implements the MD and the mobile unit used to conduct the manual site surveys.

#### 4.1.1. Monitoring Devices

Ideally, the device used for continuous data collection in the long term should be low-cost and use little power, because scanning the radio environment does not require much computing power, and since it is necessary to deploy several MDs, the cost of the device should be as low as possible. The Raspberry Pi (RPi) is suitable for this task, since it is a single-board computer with a full operating system and it has an integrated Wi-Fi interface; hence, it can collect Wi-Fi samples and send them to a server easily. In addition, the RPi is low-cost and small enough to be installed in virtually any place of the building, as long as it is connected to a power supply. The cost of the RPi varies depending on the model, since each model has different specifications for the CPU, memory, and connectivity options. The Raspberry Pi Zero W is one of the less expensive models and costs around 12€ and has an integrated Wi-Fi interface. The Raspberry Pi 3B+ has more computing power than the Raspberry Pi Zero W, an integrated IEEE 802.11 b/g/n/ac wireless unit, and it costs around 38€. (Price at the time of purchase (at the beginning of 2019). Lately, the prices of the RPi devices have been increasing significantly due to supply shortages). We opted to use the RPi 3B+ as the Monitoring Device in these experiments, since it has more computing power, is easier to configure and has more connectivity options (Ethernet and USB ports).

Raspbian (also known as Raspberry Pi OS (https://www.raspberrypi.com/software/operating-systems/ (accessed on 6 June 2022))) is the official operating system of the RPi; it is a Debian-based operating system optimized for the RPi’s hardware. In this operating system, Wi-Fi samples can be obtained from the Wi-Fi interface with the iwlist command; for example, the command "sudo iwlist wlan0 scanning" allows one to obtain the information regarding the detected APs from 2.4 and 5 GHz WLANs. The result of the command includes several parameters for each detected AP, namely:

- Service Set Identifier (SSID)—the name of the network that is broadcast by the AP;
- Basic Service Set Identifier (BSSID)—the basic service set identifier of the AP;
- Received Signal Strength Indicator (RSSI)—the signal strength value in dBm;
- Channel—the frequency channel of the AP, from the 2.4 GHz or 5 GHz band;
- Link quality—aggregate value that represents the overall quality of the link.

**Figure 3:**
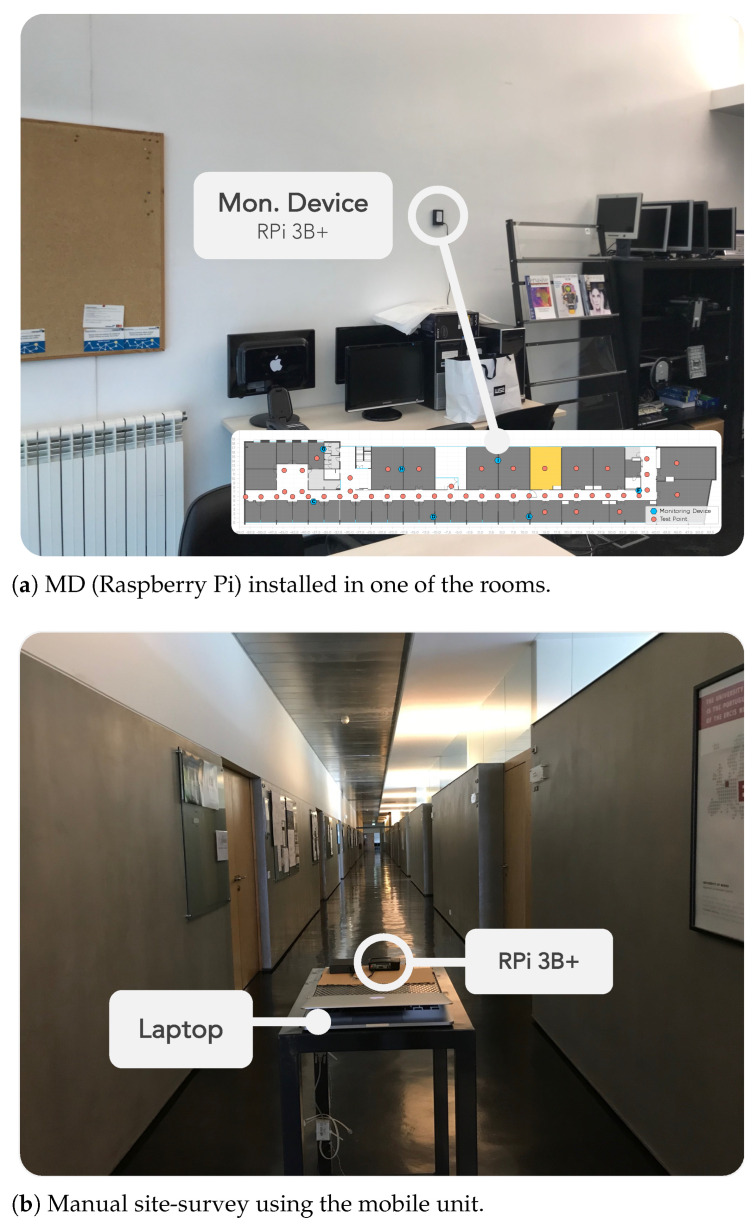
Experimental scenario.

Figure 1 shows the locations where MDs were installed in the building to provide coverage of that entire area. The selected places for the device installation are controlled environments, either offices or research laboratories. People working in these facilities were informed about the purpose of the experiment. The devices are installed in places where they do not disturb the working environment of people inside the room (Figure 3a).

Each monitoring device can be remotely configured in a safe way, using the Secure Shell (SSH) protocol. In addition, the SSH File Transfer Protocol (SFTP) can be used to safely send updated versions of the application to the MD.

The MDs were configured to collect a Wi-Fi sample every 60 s. This is an adequate value to detect small signal variations that occur during the day and more severe variations that occur over longer periods. In order to prevent the operating system from returning outdated values, two scans of the radio environment are performed. The first is discarded, and the second one is returned as the true Wi-Fi sample.

After the installation, the MDs collect data continuously 24/7 and require an Internet connection to send the collected data to the server, although it does not need to be permanent because the device is able to store data locally and send it to the server once an Internet connection is established.

#### 4.1.2. Mobile Unit

Figure 3b depicts the mobile unit used to facilitate the manual site surveys. It is a manually pushed trolley, with a RPi 3B+ connected to a portable charger (power bank) and a laptop used for remote access and control of the RPi. The user accesses the RPi via SSH and launches the site survey application (see Section 4.3). The application allows the user to annotate RP, where the mobile unit is located and allows the collection of multiple Wi-Fi samples in that position. The manual site survey is completed after repeating this process for all RPs.

### 4.2. Software for the Automatic Long-Term Data Collection

The software that enables the data collection with a set of MDs is described in this section. It comprises multiple features to ensure its operability, such as the alert when there is a problem with the data collection (e.g., when an MD stops working).

**Figure 4:**
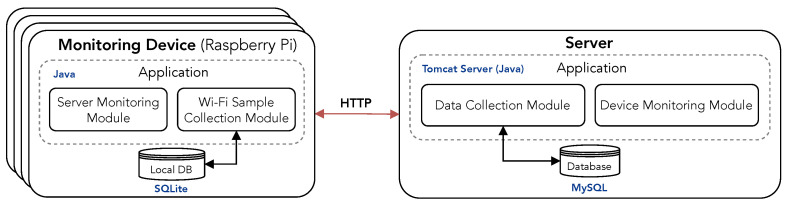
Architecture of the solution for the long-term collection of Wi-Fi samples.

A client–server application was developed to ensure that the data collection works in the long term and reports to the user whenever it is necessary to take any action to resume normal operation. The architecture of this solution, shown in Figure 4, includes the main modules and how they are connected. The Monitoring Device application is responsible for the collection of Wi-Fi samples and for sending them to a server. The server is responsible for gathering Wi-Fi samples from all MDs and storing them in a database.

#### 4.2.1. Monitoring Device Application

Each MD runs an application that includes two sub-modules: one responsible for obtaining Wi-Fi samples and saving them to a local database, and another responsible for monitoring the connection with the central server. Upon starting the application, a configuration file is loaded. Then, both modules are initialized and run in parallel. Several parameters may be set in the configuration file of the application, namely:

- Wi-Fi sample polling—the time interval between consecutive scans to obtain Wi-Fi samples, defined in seconds;
- location coordinates—latitude and longitude coordinates of the location where the MD is deployed;
- location description—textual description of the location where the MD is deployed;
- e-mail—the email address to send alerts;
- number of unsent samples—an alert email will be sent when the number of unsent Wi-Fi samples is higher than this value.

The Server Monitoring Module checks the number of unconfirmed Wi-Fi samples (to be sent to the server) and sends an email alert when the number of unsent samples is higher than the value specified in the configuration file. A new email is sent every 8 h in case it is not possible to establish a connection to the server.

The Wi-Fi Sample Collection Module performs scans of the radio environment according to the polling time defined in the configuration file. A new Wi-Fi sample is returned from the scan of the radio environment, and then it is sent to the server and stored in a local database. If the connection to the server fails, the Server Monitoring Module will check when it is possible to establish a connection to the server, and when possible the Wi-Fi samples are sent.

The Local Database is an SQLite file that stores a local backup of the collected data in each MD. The long-term collection of Wi-Fi data will lead to significant use of storage. For instance, considering the collection of samples every 60 s during one month represents a total of 43 200 Wi-Fi samples that occupy around 95 MBytes in the SQLite database file. Since queries in SQLite databases take longer as the database size increases, a new database is created every month in order to guarantee that the SQLite performance is not affected over time. This segments the collected data for each month during which the MD is operational.

#### 4.2.2. Server Application

The Server Application comprises two sub-modules, namely, the Data Collection Module, which receives and stores the Wi-Fi samples, and the Device Monitoring Module, which sends alerts via email whenever an MD has stopped sending data.

To properly configure the Device Monitoring Module, several parameters should be set in a configuration file:

- e-mail—the destination email address to whom alerts will be sent;
- time without receiving data from device (hours)—when the MD stopped sending new Wi-Fi samples over this number of hours, an email alert will be sent;
- polling time for device monitoring (seconds)—time interval in which the Device Monitoring Module checks the latest device’s connections.

#### 4.2.3. Data Collection Module

The server includes a RESTful web interface that is used by the MDs to send Wi-Fi samples. Since MDs are connected to the Internet, they can easily send data to the server through this interface, which receives the Wi-Fi samples on an HTTP request. Upon receiving a new Wi-Fi sample, the Data Collection Module processes it and stores it in a MySQL database; hence, samples from all MDs are properly stored.

#### 4.2.4. Device Monitoring Module

In order to guarantee that all monitoring devices are working properly, the Device Monitoring Module sends email alerts whenever a monitoring device has stopped sending samples to the server. The database stores the last time that the device sent data. When a certain period of time has gone by since the last connection from the device, an alert is sent via email. The number of hours that trigger an alert can be set in the configuration file of the application. The last connection of each device is checked at regular intervals by the Device Monitoring Module.

### 4.3. Software for the Manual Site Surveys

The long-term dataset is complemented with manually collected data in known positions. The main advantage of manual site surveys is that it is possible to collect data in many test locations (RPs) which would not be feasible with one MD for each RP. As a consequence, the manual site surveys depend on a user to collect a few Wi-Fi samples at each testing point, thereby representing the radio environment for a very short time window. To achieve this, an adapted version of the application described in Section 4.2.1 was used to perform the manual site surveys, which allows setting the number of Wi-Fi samples to collect at each RP and assigning a name to each position where samples are collected. This is later used to cross-reference the id of the RP to the coordinates of that point.

## 5. Database Description

The continuous data collection started on 19 February 2019 and ended after two years on 25 March 2021. The resulting dataset includes two sets of data, namely, one that includes Wi-Fi samples obtained continuously at the MDs’ positions (seven known locations) during a long time span, and another that comprises periodic site surveys at which Wi-Fi samples were collected in 49 different RPs.

Having distinct characteristics, each set of data can be used for different purposes. For example, the first set of data can be used for the interpolation of radio maps and analysis of the radio environment. The second set of data can be used as test data to validate a radio map interpolation method or for assessing fingerprinting-based IPSs.

Each dataset (either the long-term dataset or a manual site survey) is defined by four subsets of data, as follows:

(1) $$D_{q}=\left\{T,P,RSS,C\right\}$$

where *q* defines the date of the dataset in the YYYY-MM format for MDs data or YYYY-MM-DD format for site-survey data. (Formats of folder names are based on ISO 8601 [42], where YYYY defines the year (4 digits), MM defines the month (a zero-padded decimal number between 01 and 12), and DD defines the day of the month (a zero-padded decimal number between 01 and 31)). *T* comprises the set of timestamps when Wi-Fi samples were obtained, *P* represents the list of Cartesian coordinates of the points where Wi-Fi samples were obtained, RSS defines the set of Received Signal Strength (RSS) values of detected APs in Wi-Fi samples, and *C* comprises the set of frequency channels of detected APs.

The timestamps are defined as:

(2) $$T=\left\{t_{1},t_{2},\ldots ,t_{i},\ldots ,t_{N}\right\}$$

where $t_{i}$ corresponds to the timestamp of the *i*th Wi-Fi sample and is defined in the format YYYYMMDDhhmmssSSS (datetime based on ISO 8601 [42], where: YYYY correspond to the year (four digits), MM correspond to the month (2 zero-padded digits, between 01 and 12), DD correspond to the day of the month (a zero-padded decimal number between 01 and 31), hh correspond to the hour of the day (a zero-padded decimal number between 00 and 23), mm correspond to the minute (a zero-padded decimal number between 00 and 59), ss correspond to the seconds (zero-padded decimal number between 01 and 59), and SSS correspond to the milliseconds (a zero-padded decimal number between 000 and 999)).

The list of coordinates is defined as:

(3) $$P=\left\{p_{1},p_{2},\ldots ,p_{i},\ldots ,p_{N}\right\}$$

where $p_{i}=\left(x_{i},y_{i},z_{i}\right)$, which corresponds to the Cartesian coordinates of point where the *i*th Wi-Fi sample was collected. The *z* coordinate corresponds to the height considering the floor as the reference height (z=0 m).

The RSS values of a Wi-Fi sample form a list of detected APs and the respective signal strength, defined as:

(4) $$RSS=\left\{rss_{1},rss2,\ldots ,rss_{i},\ldots ,rssi_{N}\right\}$$

where $rss_{i}={\left\{AP_{1}:rssi_{1},AP_{2}:rssi_{2},\ldots ,AP_{n}:rssi_{n}\right\}}_{i}$ represents the set of signal strength values of detected APs in the *i*th Wi-Fi sample. $AP_{x}$ defines the AP’s identifier so that each detected AP has a unique id, and $rssi_{x}$ defines the signal strength value measured for that AP (in dBm).

The APs id values have the format {0,1}XXXXXXX, e.g., 100000021, where the first digit may be 1 or 0, indicating that the AP is part of the University’s WLAN infrastructure or not. These APs were identified based on the emitted SSID, being that the University’s APs usually emit the "eduroam" SSID. This information may be particularly relevant for anyone who wants to test radio map interpolation techniques, which are dependent on the APs’ information, and usually assume that APs remain in the same position. Although these APs may change due to alterations in the building’s Wi-Fi infrastructure, they have a more constant behavior than mobile hotspots or other APs that appear and disappear inside the building.

Following the same approach, the frequency channels of APs are defined as follows:

(5) $$C=\left\{c_{1},c_{2},\ldots ,c_{i},\ldots ,c_{N}\right\}$$

where $c_{i}={\left\{AP_{1}:ch_{1},AP_{2}:ch_{2},\ldots ,AP_{n}:ch_{n}\right\}}_{i}$ represents the set of channels from detected APs in the *i*th Wi-Fi sample. $AP_{x}$ defines the AP’s identifier, and the frequency channels ($ch_{x}$) can be the 2.4 GHz or the 5 GHz band.

Collecting the frequency channel of the APs allows one to perform additional studies to assess the Signal to Interference Ratio (SIR) on the building, such as to create SIR maps using interpolation, as introduced in [43,44]. Detecting the areas that are more affected by interference can be explored to reduce large errors in positioning systems. This also allows one to detect which APs are emitting in the 2.4 GHz band or in the 5 GHz band, which have different propagation characteristics due to the differences in the central frequency of channels in these bands.

### 5.1. Continuous Long-Term Dataset

The long-term dataset is composed of 7,446,538 Wi-Fi samples collected from the MDs, among which, 2711 distinct APs were detected based on the BSSID, which typically corresponds to the physical address (MAC) of the APs.

**Figure 5:**
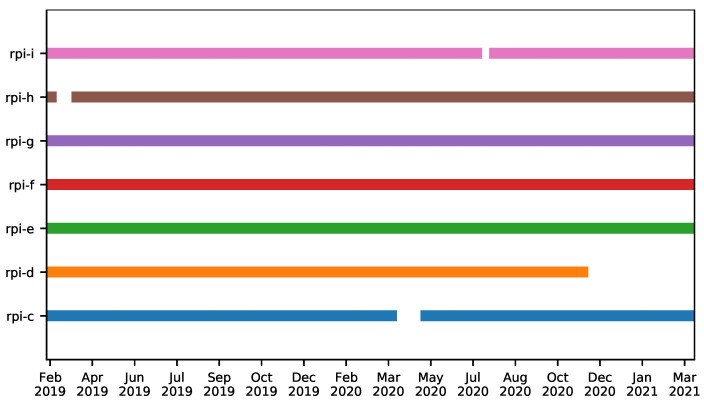
Periods during which the monitoring devices were active and collecting data.

The periods during which the MDs were operational are depicted in Figure 5. This shows times when MDs were working and collecting data. Although we tried to keep all MDs running 24/7, there were some times when they were switched off or there was an issue in the power supply, in these cases we had to physically access the device to power it on again.

**Figure 6:**
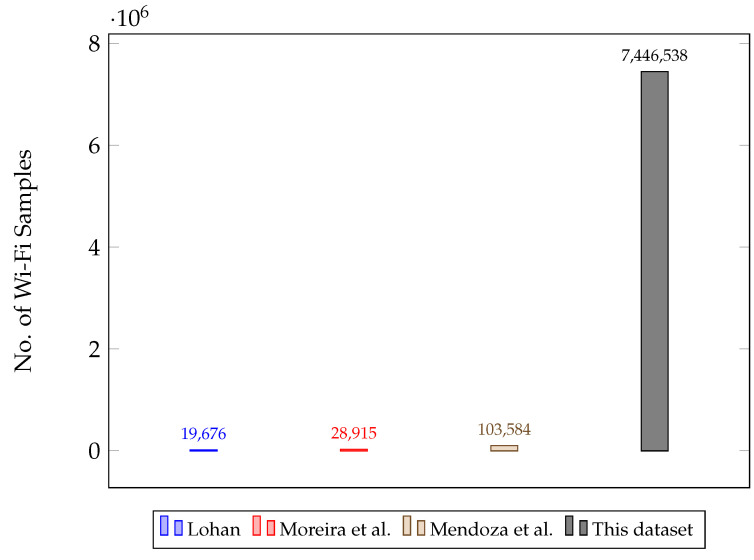
Comparison between the sizes of different Wi-Fi fingerprinting datasets.

Table 1 shows the number of Wi-Fi samples collected by each MD to have an idea of the size of the dataset. To have an idea of the size of this dataset, Figure 6 compares the number of Wi-Fi samples between several Wi-Fi fingerprinting datasets. The Lohan [8] and Moreira et al. [7] datasets have 19,676 and 28,915 Wi-Fi samples, respectively, but these are standard datasets that comprise training and test datasets collected a few days apart. The long-term dataset by Mendoza et al. [11] has a lot more samples than the previous ones with 103,584 Wi-Fi samples, but this is still far from the number of Wi-Fi samples present in this dataset, which is 7,435,398, plus the 11,140 Wi-Fi samples from the manual site surveys.

### 5.2. Manual Site Surveys

A trained person conducted manual site surveys to collect Wi-Fi data, visiting several RPs (displayed as test points in Figure 1) and collecting 20 Wi-Fi samples at each location. Just a few RPs were visited in the first site surveys, but after June 2019, almost all site surveys included samples from 49 RPs. Up until January 2020, site surveys were performed once every month, but during 2020, the university was closed from March until October due to the pandemic; hence, it was not possible to collect data during this period. Data were collected using a RPi 3 B+ placed on top of a manually pushed trolley, with a height of 1.0 m, as demonstrated in Figure 3b. While data were being collected at each RP, the person stood behind the trolley, monitoring the data collection from the laptop. There were also some times when people walked by in the corridors. Table 2 summarizes the number of surveyed RPs and the total number of Wi-Fi samples collected at each site survey.

### 5.3. Dataset Folder Structure

The dataset is divided into two main folders, one for the manual site survey datasets called site_surveys and another for the automatic and continuous long-term dataset called mon_devices. Inside the site surveys folder, each subfolder comprises the data from a site survey and is named with the naming scheme YYYY-MM-DD. Inside the continuous dataset folder, the database is divided into several subfolders. Each folder contains the measurements from MDs for the month that is defined in the subfolder name, considering the YYYY-MM naming scheme. The advantages of using several subfolders are that it allows keeping the files small, which enables faster data parsing, and it allows easy segmenting of data in case just a few selected months are considered in experiments.

Supplementary materials are included in the code folder, containing the Python scripts to parse the datasets and to create the plots included in this paper. This folder also includes the floor plan of the building used in some plots.

We adopted a similar approach to one used for similar datasets [9] to define the dataset’s structure, including the folder structure and file contents, so this dataset follows a similar format that is already used in other datasets in this area.

### 5.4. File Contents

Inside the main folder, the following files are also provided:

- coords_info.csv: list of the coordinates of RPs where Wi-Fi samples were collected on the site surveys;
- mds_info.csv: list of monitoring devices, their names, and position coordinates inside the building, where the *z* coordinate corresponds to the height of the device relative to the floor.

Each dataset, inside the subfolders mentioned above, includes four files:

- timestamps.csv: the list of timestamps as defined in *T*;
- coordinates.csv: the list of coordinates as defined in *P*;
- rssis.csv: the list of RSS values from detected APs, as defined in RSS;
- channels.csv: the list of channels from detected APs, as defined in *C*;

## 6. Data Analysis

The complementary files also provided with the dataset allow one to parse data and generate several types of plots to analyze them. The following types of plots can be created for the dataset:

- coordinates where Wi-Fi samples were collected (RPs locations in floor plan);
- Wi-Fi samples density, number of collected Wi-Fi samples at each RP, shown in the floor plan with a color scale;
- times during which MDs were operational;
- detected APs over time (global or local);
- mean Received Signal Strength Indicator (RSSI) of AP in each location, shown in floor plan;
- AP RSSI over time for a specific location;
- AP RSSI over time for all MDs;
- AP channel over time (global or local);

### 6.1. Plot Reference Points

**Figure 7:**
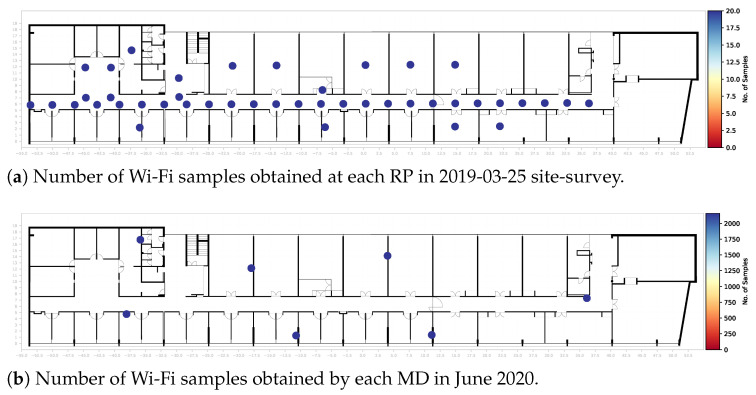
Number of Wi-Fi samples at each location.

To visualize the locations where Wi-Fi samples were collected, Figure 7a shows these locations for one of the site surveys, which also presents the number of Wi-Fi samples that were obtained at each point. Figure 7b shows the same plot for one month of data from the automatic long-term dataset.

### 6.2. Plot Monitoring Device Operational Times

Although the Device Monitoring Module checked whether the MDs were working properly, there were a few times when the MDs stopped working for some time before being switched on again. Figure 5 depicts the periods during which the MDs were working and collecting data. All MDs were operational during the majority of the time, with the exception of RPi-D, which stopped working in December 2020 due to a problem in the power supply, and it was not possible to restore it.

### 6.3. Plot AP Info over Time

**Figure 8:**
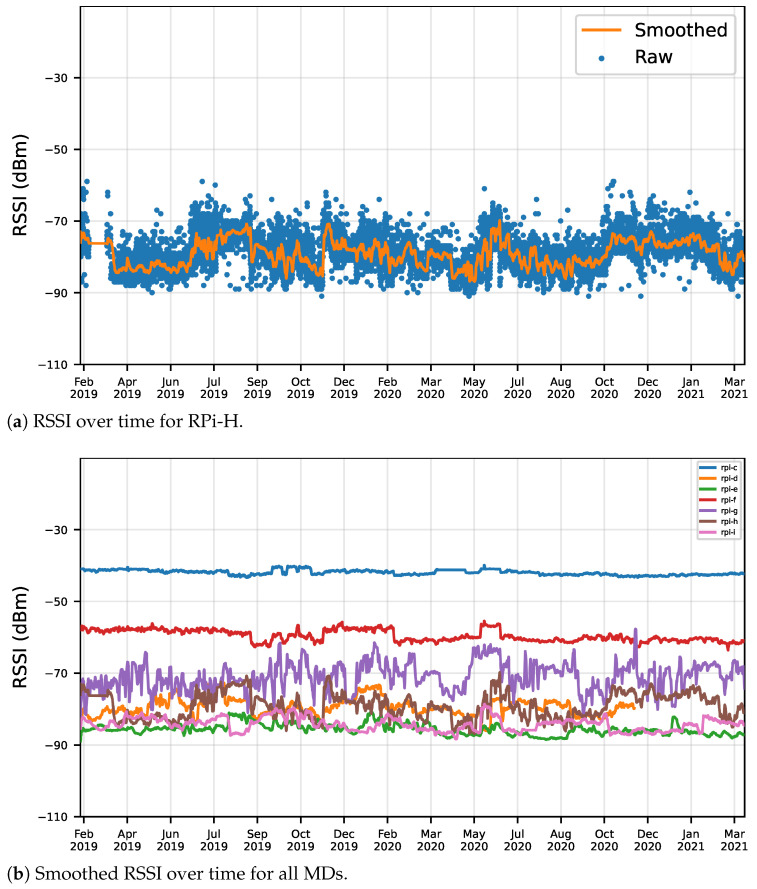
RSSI over time for AP 10000009.

The dynamic nature of APs’ signals can be observed in the signal strength variation of each AP over time, as observed by each MD. Two types of plots are provided, one that shows the raw measurements and a smoothed line (obtained from a running mean) as observed by an MD (e.g., Figure 8a), and another that plots the smoothed RSSI values for the same AP, as detected by all MDs, with a plot line representing the observed RSSI by each MD (Figure 8b). In these plots, the smoothed values are obtained from a moving average with a window size of 72 samples. As expected, the raw values in Figure 8a show that the Wi-Fi signals suffer from strong variations, and the smoothed line also shows that the mean signal of the AP also varies a lot in long term, demonstrating the dynamic nature of radio signals.

**Figure 9:**
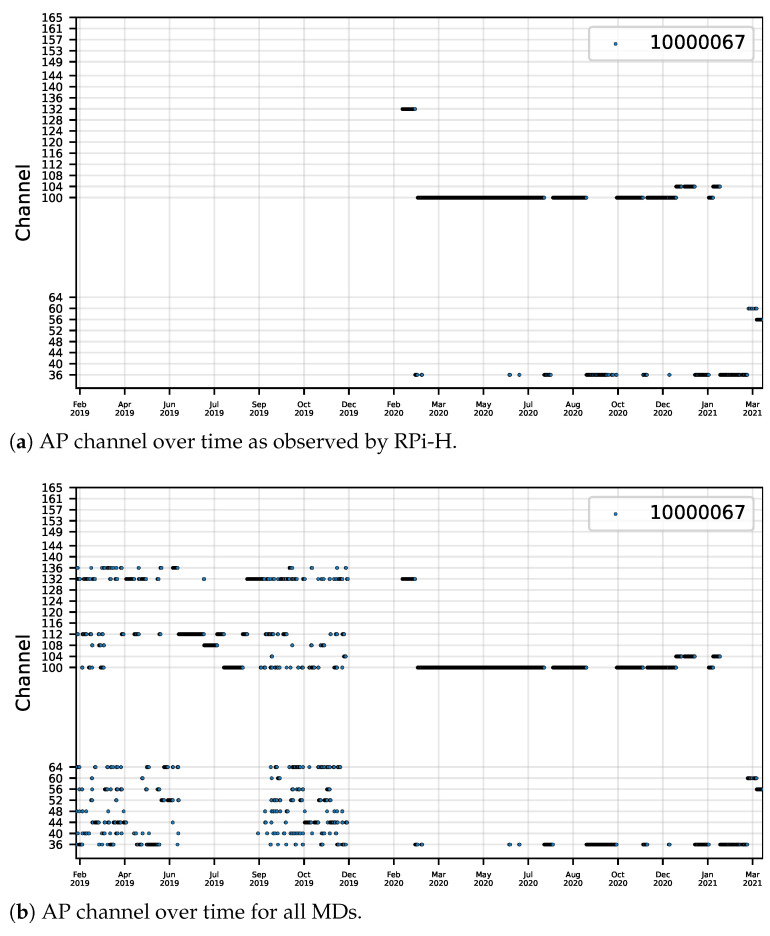
Channel over time for AP 10000067.

In addition to the significant variation in signal strength values, APs’ channels also change over time, as depicted in Figure 9, demonstrating that the Wi-Fi infrastructure is prone to changes in signal levels and the transmission frequency, which can lead to changes in the interference measured in the building. This plot has two variants, one that plots the AP’s channel from a single MD (Figure 9a), and another that plots the AP’s channel considering Wi-Fi samples from all MDs (Figure 9b). These plots are also generated for data from each month, which helps visualize the times when there are multiple channel changes near the same period.

A different set of channels is shown on the plot, depending on the AP’s transmission frequency (2.4 or 5 GHz).

### 6.4. APs Detected over Time

**Figure 10:**
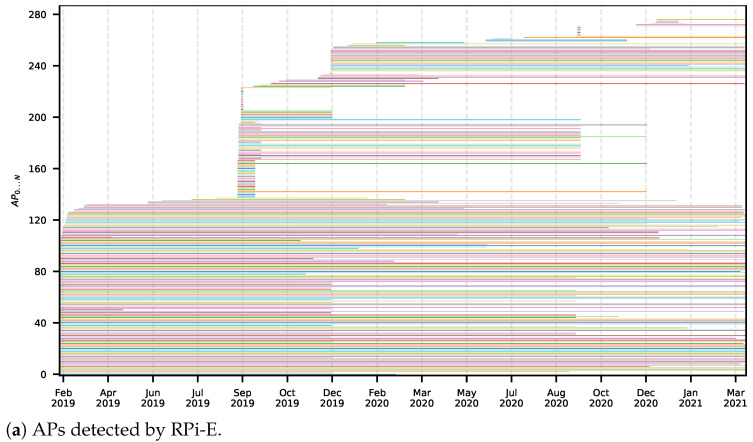

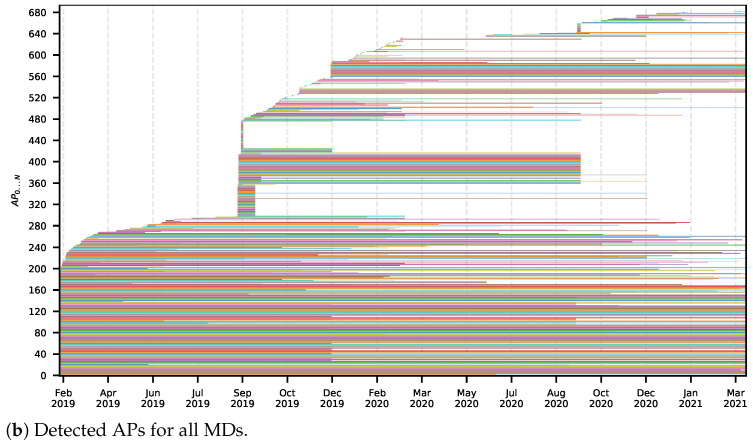
APs observed over time.

Figure 10 is a visual representation of the Wi-Fi infrastructure over time, which allows one to easily observe when significant changes occurred in the radio environment, with the removal or addition of APs. This plot has two variants, a local one which considers Wi-Fi samples from an MD (Figure 10a), and a global one which considers data from all MDs (Figure 10b), providing the perception of the radio environment of the building as observed by the MDs. As expected, the local variant shows less APs (almost 280), since it shows data only from one MD, and the global variant shows almost 680 APs detected in the building during the considered time interval. In both of these plots, we filtered out APs that are rarely observed by ignoring the ones that were detected in less than 0.01% of all Wi-Fi samples. Without this filter, a total of 2711 APs would be shown in the global plot.

## 7. Applications of the Dataset

As previously mentioned in Section 1, this dataset can be explored in several applications, e.g., analysis of long-term variations in the radio map; interpolation of radio signals to generate interpolated radio maps using techniques such as LDPL, RBF, and IDW; and for testing and evaluation of an IPS.

An application of the dataset presented in this paper is detailed in [14], where the dataset was used to measure and quantify the radio map degradation in the long term using two metrics, the positioning error with Wi-Fi fingerprinting and the radio map degradation ratio. In this study, sub-sets of the long-term dataset and the manual site surveys were used as radio maps and test data to measure the degradation over time. The positioning error with Wi-Fi fingerprinting is given by the Euclidean distance between the ground truth position and the estimated position, and the radio map degradation ratio is a novel metric that measures the variations between two radio maps. The experiments showed the variations of these metrics in a period of 2+ years. In summary, this study proved that radio maps degrade when there are significant changes in the Wi-Fi infrastructure, i.e., the addition or removal of a large number of APs.

Another application of the dataset documented in this paper is detailed in [45], where it was used with RBF to generate an interpolated radio map of the building. RBF networks are a class of artificial neural networks that were explored in [18,46] to interpolate RSS values of a radio map from Wi-Fi samples obtained at known RPs. This interpolation technique allows one to estimate signal strength values using RBF without knowledge of the indoor layout (walls, obstacles, and building materials) and without knowing the APs’ positions; therefore, it is useful, especially in situations where the building’s floor plan is not available and where there are many APs whose positions are difficult to map. Furthermore, when considering automatically collected data from MDs deployed at a few known locations, RBF allows one to construct the radio map of the building without human intervention.

Data from the MDs served as input to obtain interpolated radio maps using RBF, and then the data from the manual site surveys were used as test data to evaluate the positioning performance with Wi-Fi fingerprinting, showing a mean error ≈6 m, which is similar to the performance of other Wi-Fi fingerprinting-based positioning systems [47].

## 8. Conclusions

This paper presented a new dataset that has been published with open access, contributing to increasing the number and diversity of datasets publicly available to the community for supporting the R&D in Wi-Fi-based solutions.

A dedicated solution was devised to perform automatic data collection using several Monitoring Devices deployed in a building, continuously scanning the radio environment to obtain Wi-Fi samples. A full description of each data collection module is provided, so anyone can replicate it and collect similar long-term datasets.

The long-term continuous dataset is complemented with several site surveys, where Wi-Fi samples were manually collected throughout the building, in many reference locations. Detailed descriptions of these data were created, including the data types and how the data are organized and structured in files and folders, so that they are easy to parse and process. Complementary files are provided to facilitate this, namely, scripts to parse, process, and analyze the data, which allowed us to create the plots presented in this paper.

Several applications of the dataset were described, namely, indoor localization, analysis of the radio environment, and generating interpolated radio maps. In addition, other examples related to indoor positioning where this dataset is useful were also provided. Nevertheless, many other areas can benefit from this type of data, such as studying network deployment issues and radio-environment quality assessment.

Finally, we hope that this work will encourage other authors to share their datasets and that in the future, we may have a rich data repository available for the researchers working in this field.

## Figures and Tables

**Table 1:** Number of collected Wi-Fi samples for each Monitoring Device.

Mon. Device	No. Wi-Fi Samples
RPi-C	1,057,499
RPi-D	918,134
RPi-E	1,102,344
RPi-F	1,101,154
RPi-G	1,100,472
RPi-H	1,072,646
RPi-I	1,083,149
Total	7,435,398

**Table 2:** Manually surveyed Reference Points and number of collected Wi-Fi samples for each site survey.

Site Survey	No. RPs	No. Wi-Fi Samples
19 February 2019	26	520
25 March 2019	43	860
11 June 2019	49	980
3 July 2019	49	980
29 July 2019	49	980
11 September 2019	48	960
9 October 2019	49	980
13 November 2019	49	980
11 December 2019	49	980
15 January 2020	49	980
19 February 2021	49	980
23 April 2021	48	960
Total		11,140

## Data Availability

The dataset introduced in this paper is available in two versions: **lite version** https://doi.org/10.5281/zenodo.6646008 (accessed on 28 July 2022) which considers Wi-Fi samples from each MD every 20 min, has a total of 382,852 Wi-Fi samples, thus making it easier to parse and analyse; **full version** https://doi.org/10.5281/zenodo.6928554 (accessed on 29 July 2022) which has all collected samples, with a total of 7,446,538 Wi-Fi samples.

## Abbreviations

The following abbreviations are used in this manuscript:

AP	Access Point
BLE	Bluetooth Low Energy
BSSID	Basic Service Set Identifier
GNSS	Global Navigation Satellite System
IDW	Inverse Distance Weighted
IPIN	International Conference on Indoor Positioning and Indoor Navigation
IPS	Indoor Positioning System
LDPL	Log-distance Path Loss
MAC	Media Access Control
MD	Monitoring Device
RBF	Radial Basis Functions
RP	Reference Point
RPi	Raspberry Pi
RSS	Received Signal Strength
RSSI	Received Signal Strength Indicator
SFTP	SSH File Transfer Protocol
SIR	Signal to Interference Ratio
SSH	Secure Shell
SSID	Service Set Identifier
UWB	Ultra Wide-band
WLAN	Wireless Local Area Network
WSN	Wireless Sensor Network
